# Stomatal conductance limited the CO_2_ response of grassland in the last century

**DOI:** 10.1186/s12915-021-00988-4

**Published:** 2021-03-24

**Authors:** Juan C. Baca Cabrera, Regina T. Hirl, Rudi Schäufele, Andy Macdonald, Hans Schnyder

**Affiliations:** 1 Technical University of Munich, Lehrstuhl für Grünlandlehre, Alte Akademie 12, 85354 Freising-Weihenstephan, Germany; 2 Rothamsted Research, Sustainable Agriculture Sciences Department, Harpenden, Hertfordshire, AL5 2JQ UK

**Keywords:** ^13^C discrimination, Grassland, Hay yield, Last-century climate change, N and P nutrition status, Oxygen isotope composition of cellulose, Park Grass Experiment, Plant functional groups, Stomatal conductance, Water use efficiency

## Abstract

**Background:**

The anthropogenic increase of atmospheric CO_2_ concentration (*c*_a_) is impacting carbon (C), water, and nitrogen (N) cycles in grassland and other terrestrial biomes. Plant canopy stomatal conductance is a key player in these coupled cycles: it is a physiological control of vegetation water use efficiency (the ratio of C gain by photosynthesis to water loss by transpiration), and it responds to photosynthetic activity, which is influenced by vegetation N status. It is unknown if the *c*_a_-increase and climate change over the last century have already affected canopy stomatal conductance and its links with C and N processes in grassland.

**Results:**

Here, we assessed two independent proxies of (growing season-integrating canopy-scale) stomatal conductance changes over the last century: trends of δ^18^O in cellulose (δ^18^O_cellulose_) in archived herbage from a wide range of grassland communities on the Park Grass Experiment at Rothamsted (U.K.) and changes of the ratio of yields to the CO_2_ concentration gradient between the atmosphere and the leaf internal gas space (*c*_a_ – *c*_i_). The two proxies correlated closely (*R*^2^ = 0.70), in agreement with the hypothesis. In addition, the sensitivity of δ^18^O_cellulose_ changes to estimated stomatal conductance changes agreed broadly with published sensitivities across a range of contemporary field and controlled environment studies, further supporting the utility of δ^18^O_cellulose_ changes for historical reconstruction of stomatal conductance changes at Park Grass. Trends of δ^18^O_cellulose_ differed strongly between plots and indicated much greater reductions of stomatal conductance in grass-rich than dicot-rich communities. Reductions of stomatal conductance were connected with reductions of yield trends, nitrogen acquisition, and nitrogen nutrition index. Although all plots were nitrogen-limited or phosphorus- and nitrogen-co-limited to different degrees, long-term reductions of stomatal conductance were largely independent of fertilizer regimes and soil pH, except for nitrogen fertilizer supply which promoted the abundance of grasses.

**Conclusions:**

Our data indicate that some types of temperate grassland may have attained saturation of C sink activity more than one century ago. Increasing N fertilizer supply may not be an effective climate change mitigation strategy in many grasslands, as it promotes the expansion of grasses at the disadvantage of the more CO_2_ responsive forbs and N-fixing legumes.

**Supplementary Information:**

The online version contains supplementary material available at 10.1186/s12915-021-00988-4.

## Background

Atmospheric CO_2_ increase and related climate change are altering fundamentally the terrestrial biogeochemical cycles of carbon (C), water, and nitrogen (N) across grasslands, forests, and croplands [1–5]. At local scales, these cycles are linked by processes at plant surfaces of leaves [6–12] and roots [3, 13, 14], critical interfaces in the soil-plant-atmosphere continuum. The uptake of CO_2_ by photosynthesis and evaporative loss of water vapor in transpiration occur through the same path, the stomata [8]. These small pores in the leaf epidermis adjust their conductance in response to environmental conditions [9, 10], such as atmospheric CO_2_ concentration (*c*_a_) [7, 11], and internal cues that include photosynthetic activity [7, 15–17] which correlates with leaf nitrogen status [7, 15, 16, 18]. When stomata open for CO_2_ uptake, they simultaneously expose the leaf internal moisture to a comparatively dry atmosphere. This leaf-to-air vapor pressure gradient drives transpiration [10]. The ratio of canopy-scale photosynthesis (*A*) and transpiration (*E*) determines vegetation water use efficiency (*W*) (Eq. 1) [12, 19]. The control function of stomatal conductance, and its implication for changes of water use efficiency (*W*) in the face of increasing *c*_a_, becomes evident when *A* and *E* are expressed as the products of stomatal conductance (*g*_s_) and the concentration gradients of the respective gases [12, 19]:

1 $$ \begin{aligned} W = \frac{A}{E} = \frac{g_{s}(c_{a} - c_{i})}{g_{s} \cdot 1.6 (v_{i} - v_{a})} , \end{aligned} $$

where *c*_a_ and *v*_a_ are the CO_2_ and water vapor mole fractions in air, *c*_i_ and *v*_i_ that in the substomatal cavity, and 1.6 is the ratio of the diffusivities of CO_2_ and water vapor in air. If transpiration is altered by a change of stomatal conductance, the resulting change of the advective flux of soil water to roots may modify mass flow-dependent nutrient uptake [13, 14]. Such a change may alter the nitrogen nutrition status of vegetation [20]. Also, reduced transpiration has been identified as one of the controls limiting N acquisition in elevated CO_2_ studies in the field [3]. Understanding relationships between stomatal conductance, photosynthesis, transpiration and N acquisition are fundamental for explaining why for some temperate humid grasslands the CO_2_ fertilization responses in aboveground biomass production have been small or absent [4, 21]. That such interactions may have already operated in the last century is indicated by an investigation of herbage yields in 1891–1992 on several unlimed plots of the Park Grass Continuous Hay Experiment (Rothamsted, U.K.), which found no significant trend [22], although water use efficiency has increased [23]. It has been hypothesized from elevated CO_2_ studies in the field that the limited fertilization effect of elevated *c*_a_ in temperate grassland communities is connected with stronger reduction of stomatal conductance in the grasses, and greater photosynthetic acclimation, in comparison with forbs and legumes, or by nutrient limitation, especially for N [4, 7, 21]. Here, we examine evidence from the Park Grass Experiment to see in how far these mechanisms have already operated to shape grassland communities’ responses to the increase of *c*_a_ and associated climate change in the last century.

The Park Grass Experiment [24] (hereafter referred to as Park Grass) is the oldest permanent grassland experiment in the world. As a field resource, it enables not only exploration of the relationships between grassland community composition, nutrient status, and herbage yields, but also canopy stomatal conductance, water use efficiency, and N acquisition by ways of elemental and isotopic analysis of samples stored in the Rothamsted Sample Archive (the “Methods” section). In this analysis, we also expand on the temporal scope of earlier studies on yield trends [22] and intrinsic water use efficiency [23], by investigating changes during the last 100 years by using two periods in the early twentieth (1917–1931) and twenty-first century (2004–2018), each of 15 years. Over that time span (1917 to 2018), atmospheric CO_2_ concentration (*c*_a_) increased by c. 30% or 100 μmol mol^−1^ [25, 26].

## Results and discussion

Between periods, daily mean temperature increased by c. 1.5 °C at Rothamsted, but atmospheric vapor pressure deficit and rainfall during the main spring growth period (1 April to 30 June) did not change significantly (Additional file 1: Fig. S1).

**Fig. 1:**
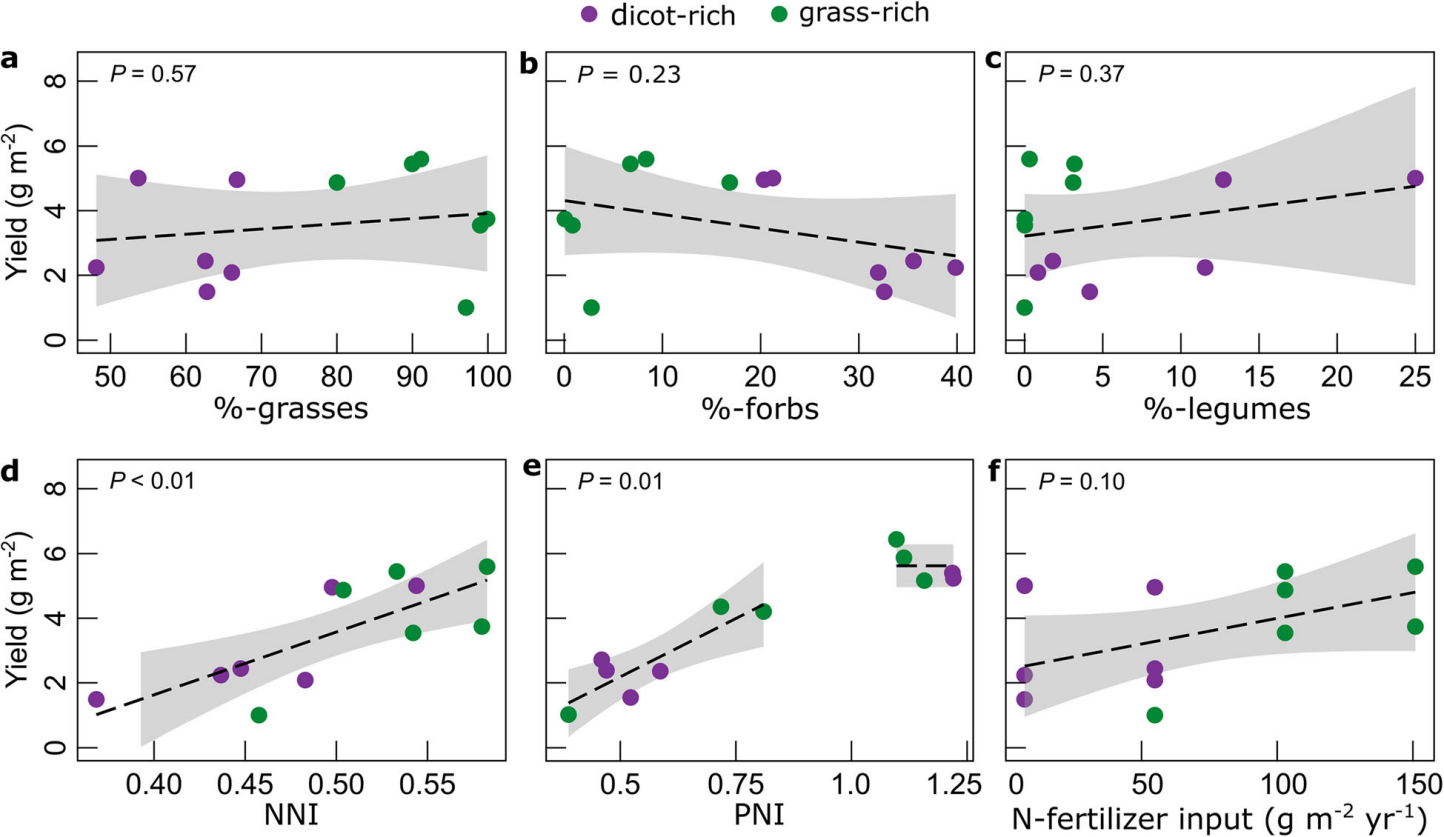
Relationship between yield and botanical traits and nutrition. Total herbage yield as a function of the percentage of grasses (**a**), percentage of forbs (**b**) percentage of legumes (**c**), nitrogen nutrition index (NNI) (**d**), phosphorus nutrition index (PNI) (**e**), and N fertilizer input (**f**). Values present the 15-year averages (2004–2018) of individual treatments, except for PNI, which was determined for samples collected between 2000 and 2009. The continuous lines and the shaded areas indicate the regression line for the relationship ± CI95%. The PNI linear regression was calculated for values < 1 only, as yields for PNI values > 1 are assumed independent of phosphorus nutrition status

We analyzed hay samples (the “Methods” section) from selected plots that had received different annual fertilizer treatments (*n* = 12) for over a century, starting in 1856. Treatments included limed and unlimed “control” plots given no fertilizer and plots receiving nitrate- or ammonia-nitrogen (N) at different rates amended with or without lime plus phosphorus and potassium (PK) fertilizer. These treatments have caused a great diversity of plant species richness [24, 27] (2–43 species) in associated grass- (80–100% grass biomass, *n* = 6 treatments) and dicot-rich communities (32–52% dicots, with 2–25% legumes, *n* = 6), and variable total herbage yields [22, 27] (100–560 g dry biomass m^−2^ year^−1^) in the 1st annual yield cut taken around mid-June each year (Table 1). High yields (yields > 80% of the highest yielding treatment) were obtained over the full range of N fertilizer application (0–14.4 g N m^−2^ year^−1^) in the limed treatments, providing that plots also received P and K fertilizers (Fig. 1).

**Table 1:** Summary characteristics of selected treatments from the Park Grass Experiment

Treatment	Plot No. (subplot)	P/K input (g m^−2^ year^−1^)	N input (g m^−2^ year^−1^)	Functional group composition (%-G/%-F/%-L)	Species richness	pH	Total herbage yield (g m^−2^)	NNI	PNI	N to P	Nutrient limitation
No nitrogen											
CONTROL.L	2/2(a), 3(a)	0/0	0	Dicot-rich (48/40/12)	43	7.2	224	0.4	0.6	11.3	Co-limitation
CONTROL.U	3(d)	0/0	0	Dicot-rich (63/33/4)	41	5.2	149	0.4	0.5	12.9	Co-limitation
PK.L	7/2(a)	3.5/22.5	0	Dicot-rich (54/21/25)	29	6.8	501	0.5	1.2	4.9	N-limitation
Sodium nitrate											
N*1.L	17(a)	0/0	4.8	Dicot-rich (62/36/2)	36	7.1	244	0.4	0.4	14.9	Co-limitation
N*1PK.L	16(a)	3.5/22.5	4.8	Dicot-rich (68/20/12)	26	6.7	496	0.5	1.2	4.8	N-limitation
N*2.PK.L	14/2(a)	3.5/22.5	9.6	Grass-rich (80/17/3)	31	6.9	487	0.5	1.2	5.2	Co-limitation
Ammonium sulfate											
N1.L	1(b)	0/0	4.8	Dicot-rich (66/33/1)	30	6.2	208	0.5	0.5	15.6	Co-limitation
N1.U	1(d)	0/0	4.8	Grass-rich (97/3/0)	6	4.1	100	0.5	0.4	20.1	Co-limitation
N2PK.L	9/2(b)	3.5/22.5	9.6	Grass-rich (90/7/3)	19	6.3	545	0.5	1.1	5.3	N-limitation
N2PK.U	9/2(d)	3.5/22.5	9.6	Grass-rich (99/1/0)	5	3.7	355	0.5	0.8	7.9	N-limitation
N3PK.L	11/1(b)	3.5/22.5	14.4	Grass-rich (91/9/0)	16	6.2	560	0.6	1.1	6.0	N-limitation
N3PK.U	11/1(d)	3.5/22.5	14.4	Grass-rich (100/0/0)	2	3.6	374	0.6	0.7	9.9	Co-limitation

Lime application is represented with the letters “L” (limed) or “U” (unlimed) in the treatment name. Ground chalk was applied as necessary to maintain soil at pH 7 or 6 on sub-plots “a” and “b”, respectively; P amount applied to the plots was decreased from 3.5 to 1.7 g P m^−2^ year^−1^ since 2017; functional group composition was estimated from botanical separation data between 1915 and 1976, 1991 and 2000, and 2010 and 2012 (e-RA); species richness data report on a 10-year period from 1991 to 2000; total herbage yields and nitrogen nutrition index (NNI) data are given for the last 15 years of the study (2004–2018); phosphorus nutrition index (PNI) and N to P ratios are presented for a 10-year period (2000–2009); soil pH corresponds to samples taken in 1995; NNI was calculated as in Lemaire et al. [28], with parameters for C3 temperate grassland if yield > 100 g m^−2^ and N_crit_ = 4.8%-N in dry matter if yield < 100 g m^−2^. PNI was calculated according to Liebisch et al. [29]. Nutrient limitation was defined based on N to P ratios according to Güsewell [30], with N to P ratios < 10 and > 20 corresponding to N- and P-limitation and N to P ratios between 10 and 20 indicating N and P co-limitation

We determined intrinsic water use efficiency (*W*_i_), the physiological, i.e., non-climatic component of water use efficiency, that accounts separately for atmospheric water demand (*v*_a_ – *v*_i_) [12, 19]:

2 $$ \begin{aligned} W_{i} = W(v_{a} - v_{i}) = \frac{A}{g_{s}} = \frac{c_{a} - c_{i}}{1.6} = \frac{c_{a} (1 - \frac{c_{i}}{c_{a}})}{1.6} .
\end{aligned} $$

**Fig. 2:**
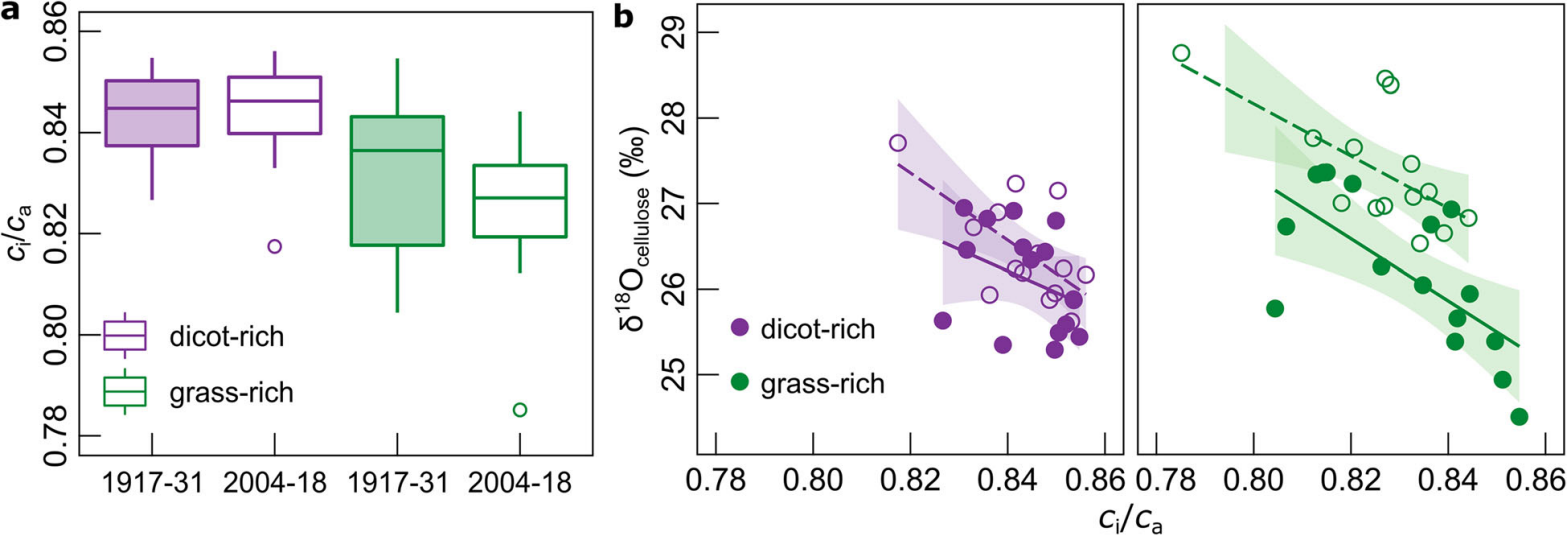
*c*_i_/*c*_a_ variation during the last century. *c*_i_/*c*_a_ variation for 15-year periods at the beginning (1917–1931) and the end (2004–2018) of the last century (**a**) and its relationship with δ^18^O_cellulose_ variation (**b**). *c*_i_/*c*_a_, the ratio of substomatal to atmospheric CO_2_ concentration, was calculated from the C isotope composition of hay samples using the model of Ma et al. [28] (the “Methods” section). Values represent the yearly averages of samples during the two periods of analysis (1917–1931, filled symbols; 2004–2018, empty symbols). The lines in **b** (1917–1931, continuous line; 2004–2018, dashed line) and the shaded areas indicate the regression line for the relationship ± CI95%

**Fig. 3:**
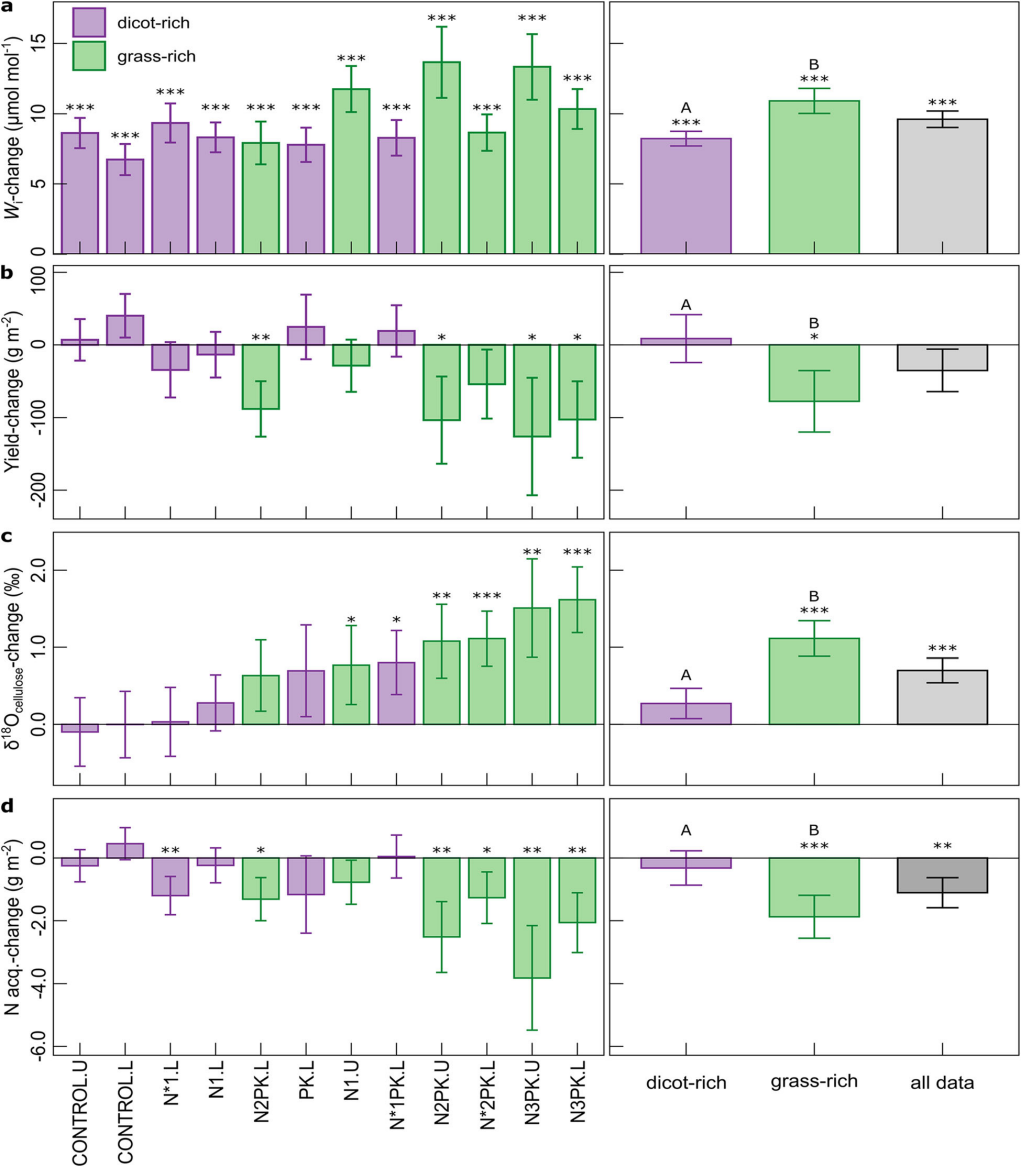
Long-term changes in physiological parameters at the Park Grass Experiment. Intrinsic water-use efficiency-change (*W*_i_-change) (**a**), hay yield-change (**b**), δ^18^O_cellulose_-change (**c**), and nitrogen acquisition-change (**d**) during the last century. Changes were calculated as the difference between period 2 (2004–2018) and period 1 (1917–1931). Results are presented for each fertilizer treatment (in the order of the treatment effect on the long-term δ^18^O_cellulose_-change), the mean of all dicot-rich and grass-rich treatments, and all treatments combined. Bar plots and error bars represent the calculated difference ± SE (SE calculated as SE from period 1 + SE from period 2; *n* = 11–15 in each period for each treatment depending on data availability; see Additional file 1: Table S1 for details). *t* tests were used for determining if the long-term changes were statistically significant and significance is presented when *P* < 0.05. Significance levels: *P < 0.05; **P < 0.01; ***P < 0.001. Significant differences between grass-rich and dicot-rich treatments are designated with different capital letters

*c*_i_/*c*_a_ was determined from ^13^C discrimination (the “Methods” section), in accounting for effects of mesophyll conductance and photorespiration [31], factors that were not previously considered [23]. Equations 1 and 2 show that water use efficiency depends principally on photosynthesis and stomatal conductance, if atmospheric water demand is constant, as was approximately the case at Park Grass (Additional file 1: Fig. S1). Additionally, water use efficiency increases in proportion to *c*_a_ if 1 − *c*_i_/*c*_a_, the relative CO_2_ concentration gradient between the atmosphere and the leaf intercellular space, does not vary. Our results demonstrated that *c*_i_/*c*_a_ did not change significantly between the start and the end of the century in the dicot-rich communities, but decreased by 0.01 in grass-rich communities (*P* < 0.01). Besides, *c*_i_/*c*_a_ was slightly higher (and varied less within periods) in dicot-rich relative to grass-rich treatments, both at the beginning (+ 0.01, *P* < 0.001) and at the end of the century (+ 0.02, *P* < 0.001) (Fig. 2). The modest observed variation of *c*_i_/*c*_a_ across the century agrees with other ^13^C-based observations over geological timescales with greatly varying *c*_a_ [11] and with plant responses from seasonal- to century-scale changes of climatic, edaphic, and nutritional stresses in forests and grasslands, including at Park Grass [5, 23, 32]. Modest variation of *c*_i_/*c*_a_ is an expected result of coordinated reciprocal adjustments of photosynthesis and stomatal conductance that serve to optimize photosynthetic C gain relative to water loss by transpiration [5, 12, 18, 33]. On average, intrinsic water use efficiency at Park Grass increased by + 9.6 μmol mol^−1^ or c. 31% compared to the beginning of the century (Fig. 3a, *P* < 0.001), mainly due to the increase of *c*_a_. However, the increase of intrinsic water use efficiency was c. 33% greater in the grass-rich swards than dicot-rich plant communities, due to the small decrease of *c*_i_/*c*_a_.

Yield trends over the century diverged for the dicot- and grass-rich treatments (Fig. 3b, *P* < 0.001). While the yields of the grass-rich treatments decreased by 16% on average, yield trends of the dicot-rich plots and the average of all plots were not significant, aligning with the previous observation that annual yields of the Park Grass plots have not increased systematically in the twentieth century [22]. Changes of yield (*Y*) are linked to changes of growing season-integrated water use efficiency via canopy photosynthesis as *Y* = *A* (1 –*ϕ*) (1 – *r*) [19], with *ϕ* the proportion of carbon respired and *r* that allocated to roots and non-harvested or senesced shoot biomass. Growing season-integrated canopy photosynthesis of the different treatments may have changed over the century in response to rising *c*_a_, warming (and associated earlier spring growth) and precipitation patterns and weather dynamics in intricate but unknown ways. Also, there is some uncertainty about long-term and treatment effects on *ϕ* and *r* (but see the “Methods” section). Interestingly, however, yield trends were closely negatively related to intrinsic water use efficiency (*R*^2^ = 0.53; *P* < 0.01). That negative relationship points to stomatal conductance (integrated over the growing season and canopy) as the likely primary control of treatment-dependent yield trends in the last century.

**Fig. 4:**
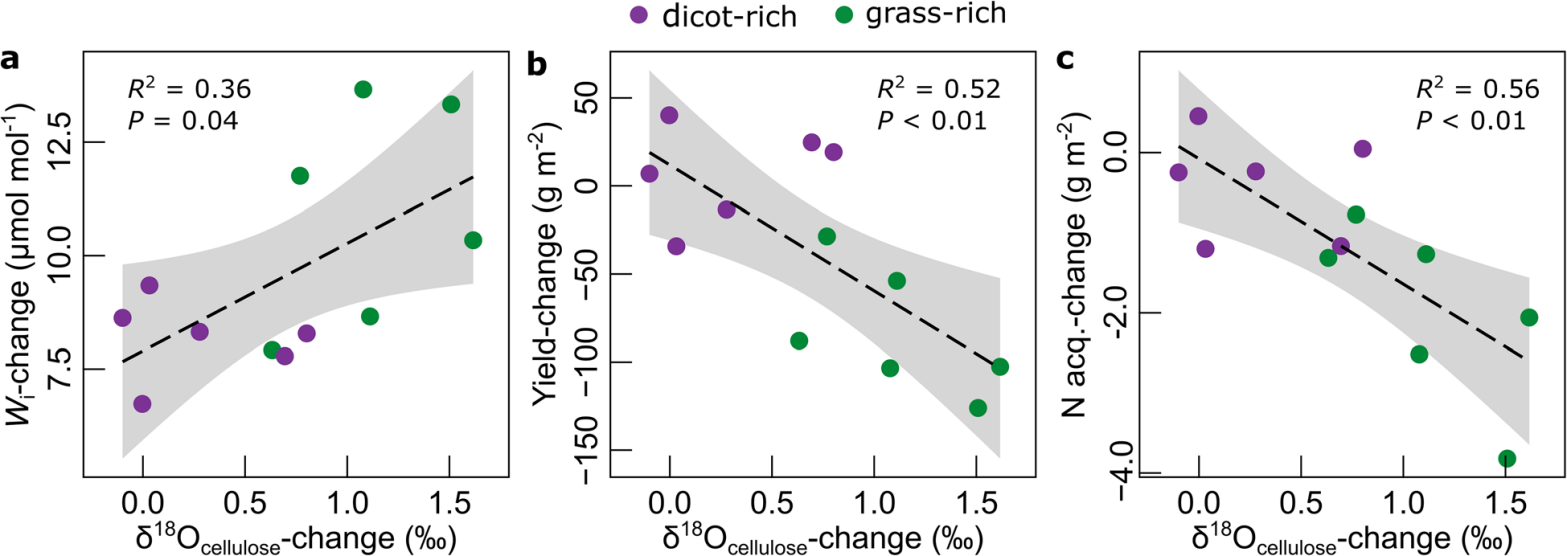
Physiological correlates of long-term δ^18^O_cellulose_-change: relationship between long-term change in δ^18^O_cellulose_ and change in intrinsic water use efficiency (*W*_i_) (**a**), hay yield (**b**), and nitrogen acquisition (**c**), for individual treatments. Long-term changes were calculated as in Fig. 3. The dashed lines and the shaded areas represent the regression line ± CI95%

**Fig. 5:**
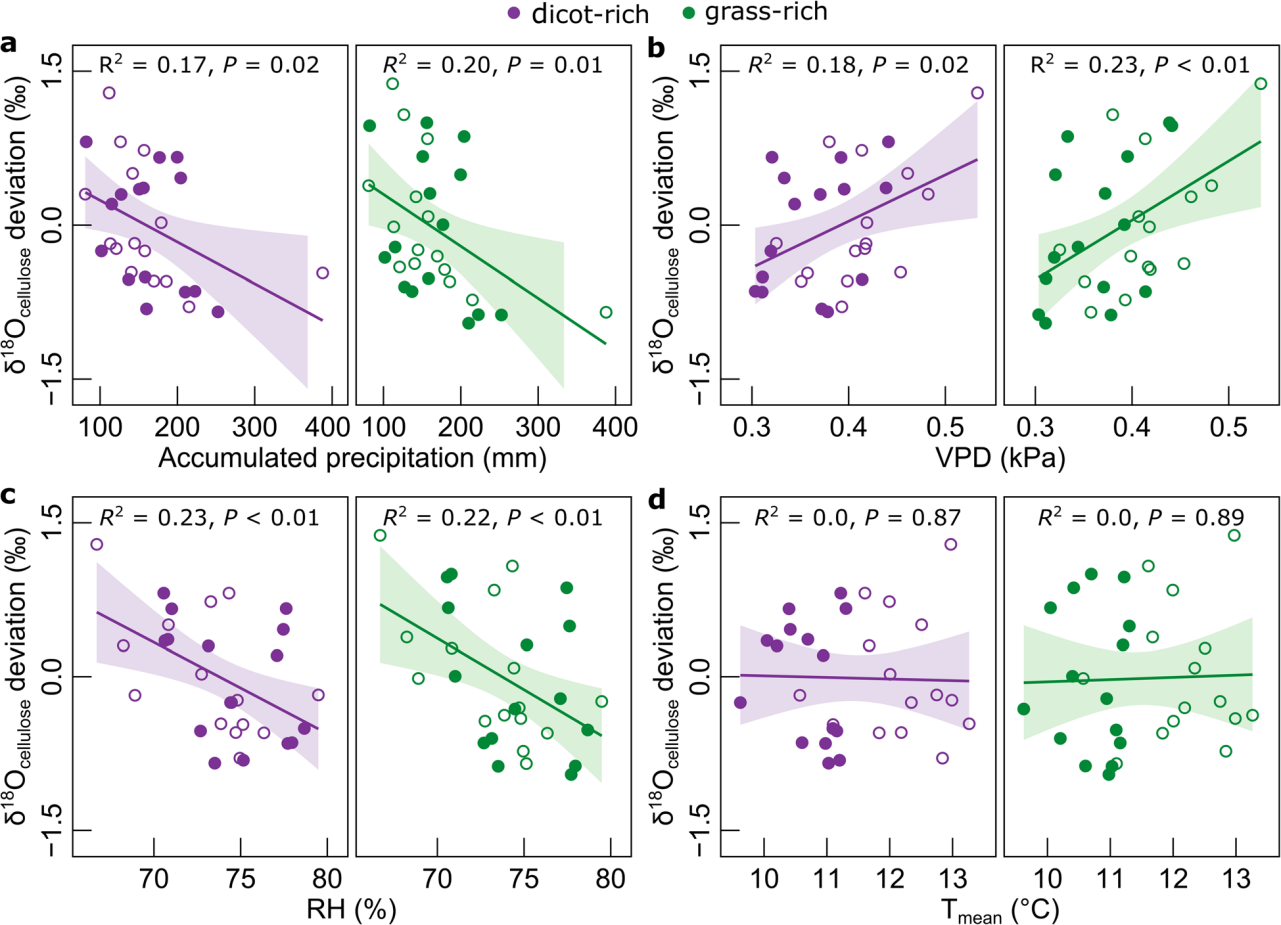
Effect of spring meteorology on δ^18^O_cellulose_. Relationship between δ^18^O_cellulose_ deviation from period mean and accumulated annual precipitation (**a**), average vapor pressure deficit (VPD) (**b**), average relative humidity (RH) (**c**), and average temperature (**d**) during the spring growing period (1 April to 30 June). δ^18^O_cellulose_ deviation values represent the difference between yearly averages of all samples from grass-rich or dicot-rich treatments and their respective 15-year averages during period 1 (1917–1931, filled circles) and period 2 (2004–2018, empty circles). The continuous lines and the shadowed areas indicate the regression line for the relationship ± CI95%

Indeed, treatment effects on yield trends also correlated negatively with trends of δ^18^O of cellulose (δ^18^O_cellulose_, the “Methods” section) (Fig. 4b, *R*^2^ = 0.52, *P* < 0.01). Increases of δ^18^O_cellulose_ in plant biomass are indicative of decreases of stomatal conductance, under otherwise equal environmental conditions [34, 35], including rainfall, atmospheric humidity, soil conditions affecting plant-water relations, and isotopic inputs of rain (δ^18^O_rain_) and atmospheric moisture (δ^18^O_vapour_) (the “Methods” section). Climate warming should increase δ^18^O_rain_ [36] and has caused an increase of vapor pressure deficit in Europe, which contributes to reduce stomatal conductance [37]. Yet, vapor pressure deficit and rainfall during spring growth have not changed significantly at Park Grass. Secondly, measurements at a 55 km-distant station (Wallingford) and predictions from global circulation models provide no indication for significant trends of annual δ^18^O_rain_ (Additional file 1: Fig. S2). And thirdly, the long-term trends of δ^18^O_cellulose_ were independent of interannual variations of meteorological variables (such as vapor pressure deficit), which were very similar at the beginning of the 20th and the twenty-first century (Fig. 5). Most importantly, the century-scale divergence of δ^18^O_cellulose_ between treatments was not attributable to eventual changes of δ^18^O_rain_ or environmental conditions, as all treatments experienced the same site and weather conditions.

**Fig. 6:**
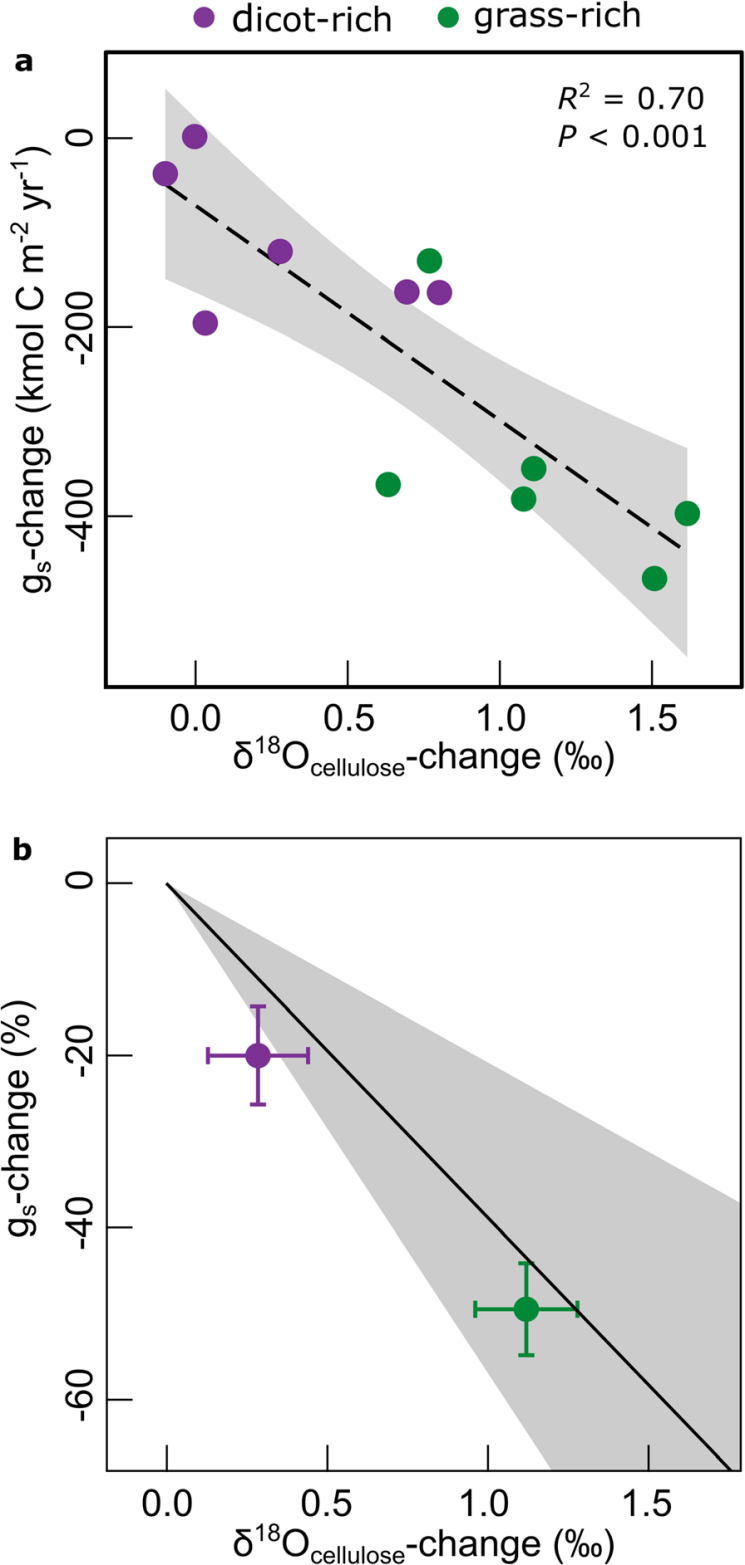
Sensitivity of stomatal conductance (*g*_s_) to δ^18^O_cellulose_. Relationship between the long-term change in δ^18^O_cellulose_ and the long-term change in stand-scale, growing season-integrated stomatal conductance, estimated as the ratio of C-yield of hay to the CO_2_ concentration gradient between the atmosphere and the leaf internal gas space (the “Methods” section) of individual treatments (**a**), and relationship between relative stomatal conductance change (in % gs change) and δ^18^O-increase (in permil) at the Park Grass Experiment for dicot-rich (*n* = 6) or grass-rich (*n* = 6) treatments (mean ± SE) compared to reports of δ^18^O_cellulose_ change versus leaf- or canopy-scale stomatal conductance change over a range of plant species or genotypes in different environmental conditions (see Additional file 1: Table S2 for details on reported studies) (**b**). The dashed line and the shaded area in **a** represent the regression line ± CI95%, while the continuous black line and the shaded area in **b** represent the mean sensitivity ± CI95% calculated from the reported studies

On average of all treatments, δ^18^O_cellulose_ increased over the century (Fig. 3c, average + 0.7‰, *P* < 0.001), consistent with a general long-term decrease of stomatal conductance. The increase of δ^18^O_cellulose_ was much stronger in grass-rich (+ 1.1‰; *P* < 0.001) than dicot-rich (+ 0.3‰; *P* = 0.06) plots. The trends of δ^18^O_cellulose_ were closely proportional (*R*^2^ = 0.70; *P* < 0.001) to trends in the ratio of hay yield to the CO_2_ concentration gradient between the atmosphere and the leaf internal gas space, *c*_a_ – *c*_i_ (Fig. 6a), another proxy of stomatal conductance integrated over the growing season and canopy (the “Methods” section). The proportionality indicated that a long-term change in δ^18^O_cellulose_ only occurred when stomatal conductance changed. Additionally, the sensitivity of δ^18^O_cellulose_ changes to changes of stomatal conductance estimated for Park Grass agreed with sensitivities observed by others (the “Methods” section) in leaf- or canopy-scale stomatal conductance studies with a range of plant species or genotypes in different environmental conditions (Fig. 6b), instilling further confidence in δ^18^O_cellulose_ change as a well-grounded measure of long-term canopy-scale stomatal conductance variation at the Park Grass site in the past century. It is also notable in that context, that interannual variation of δ^18^O_cellulose_ correlated negatively with *c*_i_/*c*_a_, both at the beginning and end of the century (*P* < 0.001), with greater ranges of variation in grass-rich than dicot-rich plots (Fig. 2b). This relationship also points to stomatal conductance-variation as an important cause for interannual variation of intrinsic WUE at Park Grass, particularly in the grass-rich plots.

Negative yield trends in grass-rich communities over the century were connected with decreased net rates of N acquisition (Fig. 3d, − 1.9 g N m^−2^ year^−1^, *P* < 0.001). Similar decreases of N acquisition were generally not observed in dicot-rich treatments (− 0.3 g m^−2^ year^−1^, *P* = 0.4), that received little or no N fertilizer. These observations match observations in elevated CO_2_ studies, which found consistently decreased rates of N acquisition in grassland, forests and crops under conditions where elevated CO_2_ failed to enhance yields [3]. The Park Grass data indicate that such *c*_a_- and climate change-induced reductions of N acquisition may have occurred mainly in grass-rich grassland communities in the last century. At Park Grass, these decreases in N acquisition were closely correlated with increases of δ^18^O_cellulose_ (Fig. 4c, *R*^2^ = 0.56; *P* < 0.01), in agreement with stomatal conductance-mediated reductions of transpiration and associated mass flow-dependent N acquisition [3, 13, 14]. Absence of significant reductions of N acquisition in the non-N-fertilized (dicot-rich) communities agrees with much smaller (if any) reductions of stomatal conductance, but may also be influenced by the fact that N acquisition in N-poor soils is more closely controlled by diffusion [14], and atmospheric deposition of N and biological N fixation account for a larger proportion of total N acquisition in these plots on average [24]. But, regardless of the mechanism underlying the reduced N acquisition [3] in grass-rich plots, any impairment of N status would reduce photosynthetic capacity and feedback on stomatal conductance to adjust *c*_i_/*c*_a_ for optimization of C gain per unit water loss by transpiration [6, 7, 10, 15, 16, 18].

N fertilizer addition was the strongest experimental determinant of grass dominance at Park Grass (*P* < 0.001). Meanwhile, PK fertilization had a much greater effect on yields, although all plots remained N limited or N and P co-limited (Table 1). N limitation [2, 20, 28] is a well-known factor limiting the CO_2_ fertilizer effect in grassland [4, 21]. During the century, the reduction of N acquisition aggravated the N limitation of the grass-rich communities, as evidenced by a decreasing nitrogen nutrition index [28] (− 0.08, *P* < 0.001). Conversely, the nitrogen nutrition index decreased much less in the dicot-rich communities (− 0.04, *P* = 0.01). That difference must have contributed to the convergence of yields of grass- and dicot-rich plots over the century. For example, among the high-yielding, limed and PK-fertilized plots, the dicot-rich, non-N-fertilized plot yielded 22% less than its grass-rich, N-fertilized counterparts at the beginning of the twentieth century, but only 6% less at the end of the twenty-first century. Thus, the yield enhancement generated by N fertilizer addition decreased by 78% over the century. At the same time, the apparent efficiency of N fertilizer uptake (net N acquisition per unit of added N fertilizer) decreased by 16% in the grass-rich (*P* < 0.001) but not in the dicot-rich communities (*P* = 0.2). Although the cutting frequency (two cuts per year) at Park Grass does not represent a very intensive utilization practice, the decreasing N fertilizer uptake efficiency of grass-rich communities does raise questions concerning the fate of N in the ecosystem and the future sustainability of high inputs of N fertilizer in such grasslands [38]. Much of the Western European grassland receives very high N inputs [39].

## Conclusions

The present work provides evidence for an interaction between plant functional groups and the *c*_a_- and related climate change-response of temperate-humid permanent grassland in the last century, managed under a hay-cutting regime with a range of fertilizer inputs. While part of that evidence is correlative, its interpretation is underpinned by empirical knowledge and mechanistic understanding obtained in more controlled experimental conditions, although these were mainly concerned with the effects of future elevated CO_2_ and changed climate [3, 6, 7, 21]. The observed interactions between plant functional group composition and climate change responses in the last century appeared to be associated primarily with the much greater *c*_a_-sensitivity of grasses in terms of their stomatal conductance compared with that of forbs and legumes, resulting in effects on grassland vegetation water use efficiency [23], N acquisition and aboveground biomass production, central features of the role of vegetation in biogeochemical cycles and feedbacks to the climate system. Our work also shows that key predictions drawn from free-air CO_2_ enrichment experiments concerning *c*_a_-saturation of the CO_2_ fertilizer effect on aboveground biomass production [4, 6, 21], N acquisition [3] and the lesser competitive ability of grasses relative to forbs and legumes [6, 21] have already taken hold in some temperate grasslands in the last century or before.

## Methods

### The Park Grass Experiment

The Park Grass Experiment (hereafter referred to as Park Grass), located at the Rothamsted Research Station in Hertfordshire, U.K., approx. 40 km north of London (0°22′ West, 51°48′ North), is the oldest grassland experiment in the world. It was started in 1856 on c. 2.8 ha of old grassland, and its original purpose was to investigate the effect of fertilizers and organic manures on hay yields of permanent grassland [40]. The experiment comprises 20 main plots with different fertilizer inputs; most plots have been divided into subplots “a”–“d” and receive different lime inputs. Since the beginning of the experiment, herbage has been cut in mid-June and made into hay (first cut). Herbage regrowth of the sward was grazed by sheep during the first 15 years, but in each year since 1875, a second cut has been taken and removed from the plot. Originally, total herbage yields were determined in situ by weighing the dried material (hay) for whole plots. Since 1960, one or two sample strips, depending on the plot size, were cut on each plot using a forage harvester. The fresh material was weighed and yields were calculated after drying. Since 1856, representative hay samples from each harvest have been stored in the Rothamsted Sample Archive [41].

For this study, we used dried hay or herbage samples from the first cut (spring growth) of 13 plots (12 different treatments), with contrasting fertilizer and lime inputs (Table 1). The treatments included four nitrogen levels (0, 4.8, 9.6, and 14.4 g N m^−2^ year^−1^, with nitrogen added either as ammonium sulfate or sodium nitrate), lime application (chalk applied as necessary to maintain soil at pH 7, 6, and 5 on sub-plots “a,” “b,” and “c”), one sodium/potassium level (3.5 g P m^−2^ year^−1^ as triple superphosphate and 22.5 g K m^−2^ year^−1^ as potassium sulfate; P was applied at 1.7 g m^−2^ year^−1^ since 2017), and the control plots (no fertilizer application). The plots that received P and K also received 1.5 g Na m^−2^ year^−1^ as sodium sulfate and 1 g Mg m^−2^ year^−1^ as magnesium sulfate. N was applied in spring and P and K in autumn.

### Functional group composition

Plant functional group (PFG) composition was strongly altered by fertilization and lime inputs on Park Grass. The original vegetation was classified post-hoc as dicotyledon-rich *Cynosurus cristatus*-*Centaurea nigra* grassland [42], but it changed rapidly following the introduction of the different treatments, creating very contrasting community compositions, which have reached a dynamic equilibrium since 1910 [41]. We consider the functional group composition (%-grasses, %-forbs and %-legumes in standing dry biomass) of each treatment to have remained relatively constant during the past 100 years. The average functional group composition was calculated from individual datasets sampled between 1915 and 1976, 1991 and 2000, and 2010 and 2012 (e-RA, http://www.era.rothamsted.ac.uk/). Given the high but variable proportion of grasses (48–100% of standing dry biomass, depending on treatment), we used this characteristic to represent the functional composition heterogeneity among treatments. Treatments with a grass proportion > 80% were termed grass-rich, and the other treatments (grass proportion 50–68%) dicot-rich (Table 1).

### Sample preparation and analysis

Representative subsamples (2–3 g) of plant material from the first cut were taken from the Rothamsted sample archive. Two 15-year periods were selected, corresponding to the beginning (1917–1931, period 1) and the end (2004–2018, period 2) of the last 100 years. The period length was selected to account for the interannual variability in δ^18^O of α-cellulose (δ^18^O_cellulose_), which is associated with interannual variation of meteorological conditions (see the “Stomatal conductance” section and Fig. 5). The subsamples were used to determine the δ^13^C of bulk plant material (δ^13^C_p_) and δ^18^O_cellulose_. Carbon and oxygen isotope composition were expressed as:

3 $$ {\updelta}^{13}{\mathrm{C}}_{\mathrm{p}}\ \left(\mathrm{or}\ {\updelta}^{18}{\mathrm{O}}_{\mathrm{cellulose}}\right)=\left(\frac{R_{\mathrm{sample}}}{R_{\mathrm{standard}}}-1\right), $$

with *R*_sample_ the ^13^C/^12^C (or ^18^O/^16^O) ratio of the sample and *R*_standard_ that in the international standard (Vienna Pee Dee Belemnite, V-PDB for carbon or Vienna Standard Mean Ocean Water, V-SMOW for oxygen isotope ratio).

α-cellulose was extracted from 50 mg of dry sample material by following the Brendel et al. [43] protocol as modified by Gaudinski et al. [44]. The extracted cellulose was re-dried at 40 °C for 24 h; 0.7 ± 0.05 mg aliquots were weighed into silver cups (size 3.3 × 5 mm, IVA Analysentechnik e.K., Meerbusch, Germany) and stored in an exsiccator vessel containing Silica Gel Orange (ThoMar OHG, Lütau, Germany) prior to analysis. Samples were pyrolyzed at 1400 °C in a pyrolysis oven (HTO, HEKAtech, Wegberg, Germany), equipped with a helium-flushed zero blank auto-sampler (Costech Analytical technologies, Valencia, CA, USA) and connected via an interface (ConFlo III, Finnigan MAT, Bremen, Germany) with a continuous-flow isotope ratio mass spectrometer (Delta Plus, Finnigan MAT). Solid internal laboratory standards (SILS, cotton powder) were run each time after the measurement of four samples. Both cellulose samples and SILS were measured against a laboratory working standard carbon monoxide gas, previously calibrated against a secondary isotope standard (IAEA-601, accuracy of calibration ± 0.25‰ SD). The precision for the laboratory standard was < 0.3‰ (SD for repeated measurements).

For measurement of δ^13^C_p_, the plant material was dried at 40 °C for 48 h, ball milled to a fine powder, re-dried during 24 h at 60 °C, and aliquots of 0.7 ± 0.05 weighed into tin cups (size 3.3 × 5 mm, LüdiSwiss, Flawil, Switzerland). Samples were combusted in an elemental analyzer (NA 1110; Carlo Erba, Milan, Italy) interfaced (Conflo III; Finnigan MAT, Bremen, Germany) with an isotope ratio mass spectrometer (Delta Plus; Finnigan MAT) and measured against a laboratory working standard CO_2_ gas, previously calibrated against a secondary isotope standard (IAEA-CH6 for δ^13^C, accuracy of calibration 0.06% SD). As a control, SILS which were previously calibrated against the secondary standard and had a similar C/N ratio as the samples (a fine wheat flour) were run after every tenth sample. The long-term precision for the SILS was < 0.2‰. C and N elemental concentration (%C and %N in dry biomass) were also measured in the same sequence. Additionally, P elemental concentration (%P in dry biomass) was determined on 20–25 mg of dry sample material for a 10-year period (2000–2009) using a modified phosphovanado-molybdate colorimetric method following acid digestion [45].

### Meteorological and yield data

Meteorology and yield datasets were obtained from the electronic Rothamsted Archive (e-RA, http://www.era.rothamsted.ac.uk/). Rainfall data have been collected continuously since 1853 at the Rothamsted Weather Station, temperature since 1873 and additional meteorological variables have been added gradually. We calculated a yearly spring value for the last 100 years (1917–2018) by averaging all daily values between 1 April and 30 June for the following meteorological variables: maximum air temperature (T_max_, °C), average air temperature (T_mean_, °C), average relative humidity of air (RH, %), and average vapor pressure deficit (VPD, kPa). In the case of precipitation (rain, mm), we used the cumulative rain amount for the same period. Yearly spring values were used for the analysis of long-term climatic changes.

Total herbage yield data from the first cut between 1917 and 2018 (expressed in g dry biomass m^−2^ year^−1^) were used to determine long-term yield trends during spring growth and for the calculation of (1) the yearly nitrogen acquisition (N acquisition, g m^−2^ year^−1^), calculated as dry matter yield × N content of dry biomass, and (2) a yield-weighted canopy stomatal conductance (see the “Stomatal conductance” section), for each of the 12 selected treatments. Given the change in the harvest method since 1960 [22, 46], we calculated an offset-correction for yield estimates pre-1960 to permit estimation of yield changes over the century on the same methodological basis. The offset-correction was based on the relationship between atmospheric CO_2_ concentration (as a long-term proxy for climate change) and measured yields from 1917 to 2018. For this, we fitted the following multiple linear regression to the data of every treatment:

4 $$ Y=\upalpha +\upbeta {\mathrm{CO}}_2+\upgamma \mathrm{period}+\upvarepsilon, $$

with *Y* denoting measured yield, β and γ the coefficients to be determined, period a dummy variable coded with 0 for yields before 1960 and 1 for yields after 1960 and ε the random error. The treatment-specific estimated γ parameter was used as the offset-correction for spring yield determinations before 1960 (Additional file 1: Fig. S3 and Table S3).

### Intrinsic water use efficiency

Intrinsic water use efficiency (*W*_i_) is a physiological efficiency [47] that represents the ratio of net photosynthesis rate (*A*) and stomatal conductance to water vapor (*g*_w_):

5 $$ {W}_{\mathrm{i}}=\frac{A}{g_{\mathrm{w}}}=\frac{c_{\mathrm{a}}-{c}_{\mathrm{i}}}{1.6}=\frac{c_{\mathrm{a}}\left(1-\frac{c_{\mathrm{i}}}{c_{\mathrm{a}}}\right)}{1.6}=\frac{c_{\mathrm{a}}}{1.6}\left[\frac{\ b-f\frac{\Gamma^{\ast }}{c_{\mathrm{a}}}-{\Delta}^{13}\mathrm{C}}{b-a+\left(b-{a}_{\mathrm{m}}\right)\frac{g_{\mathrm{s}}}{g_{\mathrm{m}}}}\right]. $$

That relationship is controlled by the CO_2_ concentration gradient between the atmosphere (*c*_a_) and the leaf internal gas space (*c*_i_), *c*_a_ - *c*_i_, and the ratio of the diffusivities of water vapor and CO_2_ (1.6) [19, 47]. The CO_2_ concentration gradient can also be expressed as the product of *c*_a_ and the relative CO_2_ concentration gradient between the atmosphere and the leaf internal gas space (1 – *c*_i_/*c*_a_) (Eq. 5). Thus, solving the equation required two parameters, *c*_a_, known from measurements of free air and air bubbles separated from ice cores [25], and *c*_i_/*c*_a_, that we estimated according to Ma et al. [31] (the bracketed term in Eq. 5). In that term, *a* is the ^13^C discrimination during diffusion of CO_2_ in air through the stomatal pore (4.4‰), *a*_m_ (1.8‰) the fractionation associated with CO_2_ dissolution and diffusion in the mesophyll, and *b* (29‰) and *f* (11‰) the fractionations due to carboxylation and photorespiration, respectively; Г* is the CO_2_ compensation point in the absence of mitochondrial respiration calculated following Brooks and Farquhar [48], and *g*_s_/*g*_m_ the ratio of stomatal and mesophyll conductance [31]. Relying on theory developed by Farquhar et al. [49] and Farquhar and Richards [19], this model accounts for the effects of mesophyll conductance and photorespiration on Δ^13^C-based estimations of intrinsic water use efficiency. Traditionally, estimations of *W*_i_ from Δ^13^C have used a more abbreviated version of the Farquhar model of photosynthetic carbon isotope discrimination (Δ^13^C) in C_3_ plants. That version neglected the photorespiration term and was based on the simplifying assumption that mesophyll conductance is infinite. However, *W*_i_ estimated from Δ^13^C using the abbreviated model systematically overestimates gas exchange-based estimates of *W*_i_, an error that can be corrected by using a constant *g*_s_/*g*_m_ ratio (0.79) based on measurements on a wide range of plant species from different functional groups (including grasses and herbaceous legumes), in moist and dry conditions [31]. Also, global scale effects of mesophyll conductance and photorespiration of C_3_ vegetation on ^13^C discrimination of the terrestrial biosphere have been evident for the last four decades of atmospheric CO_2_ increase and concurrent change of the ^13^CO_2_/^12^CO_2_ ratio [50]. Estimates of intrinsic water use efficiency at Park Grass calculated following Ma et al. [31] were closely correlated (*R*^2^ = 0.97) with those presented by Köhler et al. [23] using the abbreviated *W*_i_ model for the same treatments and time spans.

Δ^13^C was obtained from the measured δ^13^C_p_ of samples and the δ^13^C of atmospheric CO_2_ (δ^13^C_a_), as

6 $$ {\Delta }^{13}\mathrm{C}=\frac{\updelta^{13}{\mathrm{C}}_{\mathrm{a}}-{\updelta}^{13}{\mathrm{C}}_{\mathrm{p}}}{1+{\updelta}^{13}{\mathrm{C}}_{\mathrm{p}}}. $$

Estimates of *c*_a_ and δ^13^C_a_ were obtained as in Wittmer et al. [51] and Köhler et al. [52]. The time series generated by Köhler et al. [52] with *c*_a_ and δ^13^C_a_ yearly average values during spring (April–June, 1917–2009) was updated until 2018. Monthly values between 2010 and 2018 from the NOAA ESRL atmospheric stations Mauna Loa, Mace Head, and Storhofdi Vestmannaeyjar [26, 53] were used for *c*_a_ and δ^13^C_a_ estimation. Calculations of Δ^13^C and *W*_i_ were performed for each treatment, individually.

As intrinsic water use efficiency was estimated from a representative sample of a hay harvest, it represents a growing-season assimilation- and allocation-weighted measure.

### Stomatal conductance

Extrapolating from elevated CO_2_ studies [7], we hypothesized that the atmospheric CO_2_ increase had already caused partial stomatal closure in grassland vegetation during the last century. This would also contribute to explain why intrinsic water use efficiency has increased at Park Grass [23] although herbage dry matter yields have not, generally, shown a similar increase. We used two independent approaches to estimate changes of stomatal conductance at Park Grass: one based on changes of yields, atmospheric CO_2_ concentration and ^13^C discrimination, and the other on the concurrent changes of the oxygen isotope composition of cellulose, as we explain below.

Following reasoning in Farquhar and Richards [19], canopy-scale, growing season-integrated stomatal conductance to CO_2_ (*g*_s_, in mol m^−2^ s^−1^) can be estimated as:

7 $$ {g}_{\mathrm{s}}=A/\left({c}_{\mathrm{a}}\left(1-{c}_{\mathrm{i}}/{c}_{\mathrm{a}}\right)\right)=Y/\left({c}_{\mathrm{a}}\left(1-{c}_{\mathrm{i}}/{c}_{\mathrm{a}}\right)/\left(1-\phi \right)\left(1-r\right)\right), $$

with *Y* denoting yield (in moles of C in harvested biomass per ground area and per year), *ϕ* the proportion of carbon respired and *r* that allocated to roots and non-harvested shoot biomass. As measurements of *A* were unavailable, we used the yield data as a proxy and assumed that *ϕ* (0.35) and *r* (0.4) were constants across treatments and during the century. From estimates of stomatal conductance for every year, we then calculated the percent change of stomatal conductance (%*g*_s_-change) between the beginning (period 1, subscript 1) and end (period 2, subscript 2) of the century for every treatment as:

8 $$ \%{g}_{\mathrm{s}}-\mathrm{change}=\left(\left({g}_{\mathrm{s}\ 2}-{g}_{\mathrm{s}\ 1}\right)/\left({g}_{\mathrm{s}\ 2}\right)\right)\times 100. $$

Note that this procedure eliminated the constants for *ϕ* and *r*, so that the estimated %*g*_s_-changes were independent of *ϕ* and *r*, except if they had changed during the century.

For the ^18^O-based inference, theory [34, 54] and observations [35, 55–57] demonstrate that changes of stomatal conductance (*g*_s_) cause inverse changes of ^18^O-enrichment of cellulose above source water (Δ^18^O_cellulose_, with Δ^18^O_cellulose_ ≈ δ^18^O_cellulose_ − δ^18^O_source_). If the δ^18^O of source water (δ^18^O_source_) – the water taken up by the root system – is invariant, then an increase of Δ^18^O_cellulose_ implies a parallel increase of δ^18^O_cellulose_. Given that δ^18^O_source_ is determined by the δ^18^O of precipitation (δ^18^O_rain_) and plots at Park Grass receive the same precipitation, we presumed that any divergence in the δ^18^O_cellulose_ changes was independent of δ^18^O_source_ and hence attributable to changes of Δ^18^O_cellulose_. Indeed, no long-term changes were detected for δ^18^O_rain_ at Park Grass, estimated using yearly averages from the nearest monitoring station (Wallingford GNIP, c. 55 km west-southwest of Park Grass, data since 1982) [58] and outputs from the ECHAM5 global circulation model [59] for the location of the Park Grass Experiment (pixel resolution 1.125° × 1.12°, data since 1958) (Additional File 1: Fig. S2).

The effect of stomatal conductance on Δ^18^O_cellulose_ is primarily determined by three factors: (1) all oxygen in cellulose originates from water [60, 61], (2) a high proportion of that water is leaf water, which is evaporatively ^18^O-enriched during daytime and imprints its ^18^O-signal on photosynthetic products used in cellulose synthesis [62], and (3) evaporative ^18^O-enrichment of leaf water decreases with increasing stomatal conductance [63]. The δ^18^O_cellulose_ of a representative hay sample is a canopy-scale, growing season-integrated signal reflecting assimilation over the total time span that contributed substrate for cellulose synthesis in the tissues that comprise the sample [64]. However, the relation between stomatal conductance and Δ^18^O_cellulose_ is influenced by multiple morpho-physiological vegetation characteristics and environmental variables [65], so that stomatal conductance cannot be simply and quantitatively estimated from Δ^18^O_cellulose_ or δ^18^O_cellulose_ at present [66]. As no direct measurements of stomatal conductance were available at Park Grass during the last century, we compared δ^18^O_cellulose_ changes during the century with concurrent changes of stomatal conductance as estimated by Eqs. (7) and (8), to obtain an estimate of the stomatal conductance sensitivity of a unit change of δ^18^O_cellulose_. Further, we compared the δ^18^O_cellulose_ change versus %*g*_s_-change relation with published data [35, 55–57] and one unpublished data set. The published data covered a wide range of plant species or genotypes in different environmental conditions in controlled environments and in the field. The data from the graphic displays of the different studies were digitized using ImageJ [67]. In this analysis, we also included (1) the relationship between Δ^18^O_cellulose_ and canopy-scale stomatal conductance predicted by a process-based ^18^O-enabled soil-plant-atmosphere model for a multi-seasonal data set of grassland [64] and (2) an unpublished data set from monocultures of *Lolium perenne* grown at CO_2_ concentrations of 200, 400, or 800 μmol mol^−1^ in controlled environments with air temperature controlled at 20 °C:16 °C and relative humidity at 50%:75% during the 16:8 h day to night cycle, and a photosynthetic photon flux density of 800 μmol m^−2^ s^−1^ during the photoperiod [68].

Individual sensitivities for each study were calculated as the slopes of ordinary least-squares linear regressions. We then calculated a mean sensitivity ± CI95% (expressed as the decrease of stomatal conductance per unit increase in δ^18^O_cellulose_) based on the sensitivities in the individual studies (*n* = 6, Additional file 1: Table S2). Additionally, we used an analogous procedure to calculate the stomatal conductance*-*sensitivity in terms of %*g*_s_-decrease (Eq. 8) per unit increase in δ^18^O_cellulose_. In that, we took the mean stomatal conductance value of each study as the reference for calculating the %-decreases of stomatal conductance using the stomatal conductance versus δ^18^O_cellulose_ relationship of that study. On average of all the studies, stomatal conductance decreased by 39% per 1‰ increase of δ^18^O_cellulose_.

### Statistical analysis

We tested the long-term response of intrinsic water use efficiency, yield, δ^18^O_cellulose_, and N acquisition by comparing the average values of 15-year periods at the beginning (1917–1931, period 1) and the end (2004–2018, period 2) of the last 100 years. The long-term change was calculated as the difference between the averages from period 1 and 2, for (1) every treatment individually (*n* = 26–30), (2) grouping the data into dicot-rich (*n* = 164–173) and grass-rich (*n* = 161–174) treatments, and (3) combining all data together (*n* = 330–348) (see Additional file 1: Table S1 for detailed information about the sample size). *t* tests were used for testing the differences between periods. Additional *t* tests were performed to determine whether the long-term response of the analyzed variables differed between treatments with different plant functional group composition (dicot-rich vs. grass-rich communities). For this, data corresponding to period 2 were normalized by subtracting the average value from period 1, for every individual treatment (thus obtaining the net long-term change). Additionally, the relationship between the long-term changes of different variables (e.g., change in intrinsic water use efficiency vs. change in δ^18^O_cellulose_) was tested using ordinary least-squares linear regressions. Regression analysis was also used to test the effect of multiple parameters on yield. All statistical analyses were conducted in R v.4.0.2 [69]. The R package ggplot2 [70] was used for data plotting.

## Supplementary Information

**Additional file 1: Figure S1**. Spring (April–June) meteorological data during the last 100 years. **Figure S2**. δ^18^O_rain_ long-term trends. **Figure S3**. Yields at Park Grass 1917–2018. **Table S1**. Sample size for all treatments. **Table S2**. List of publications used for estimation of the average sensitivity of stomatal conductance (*g*_s_) to δ^18^O_cellulose_. **Table S3**. Pre-1960 yield offset-correction.

## Data Availability

The datasets used and/or analyzed during the current study are available from the corresponding author on reasonable request.
