# Supplementary material for: Stomatal conductance limited the CO2 response of grassland in the last century
Source: BMC Biol. 2021 Mar 24;19:50. doi: 10.1186/s12915-021-00988-4 (PMC7989024; doi:10.1186/s12915-021-00988-4)
Supplement: Supplementary file 1 — Additional file 1: Figure S1. Spring (April–June) meteorological data during the last 100 years. Figure S2. δ18Orain long-term trends. Figure S3. Yields at Park Grass 1917–2018. Table S1. Sample size for all treatments. Table S2. List of publications used for estimation of the average sensitivity of stomatal conductance (gs) to δ18Ocellulose. Table S3. Pre-1960 yield offset-correction. [file 12915_2021_988_MOESM1_ESM.pdf]

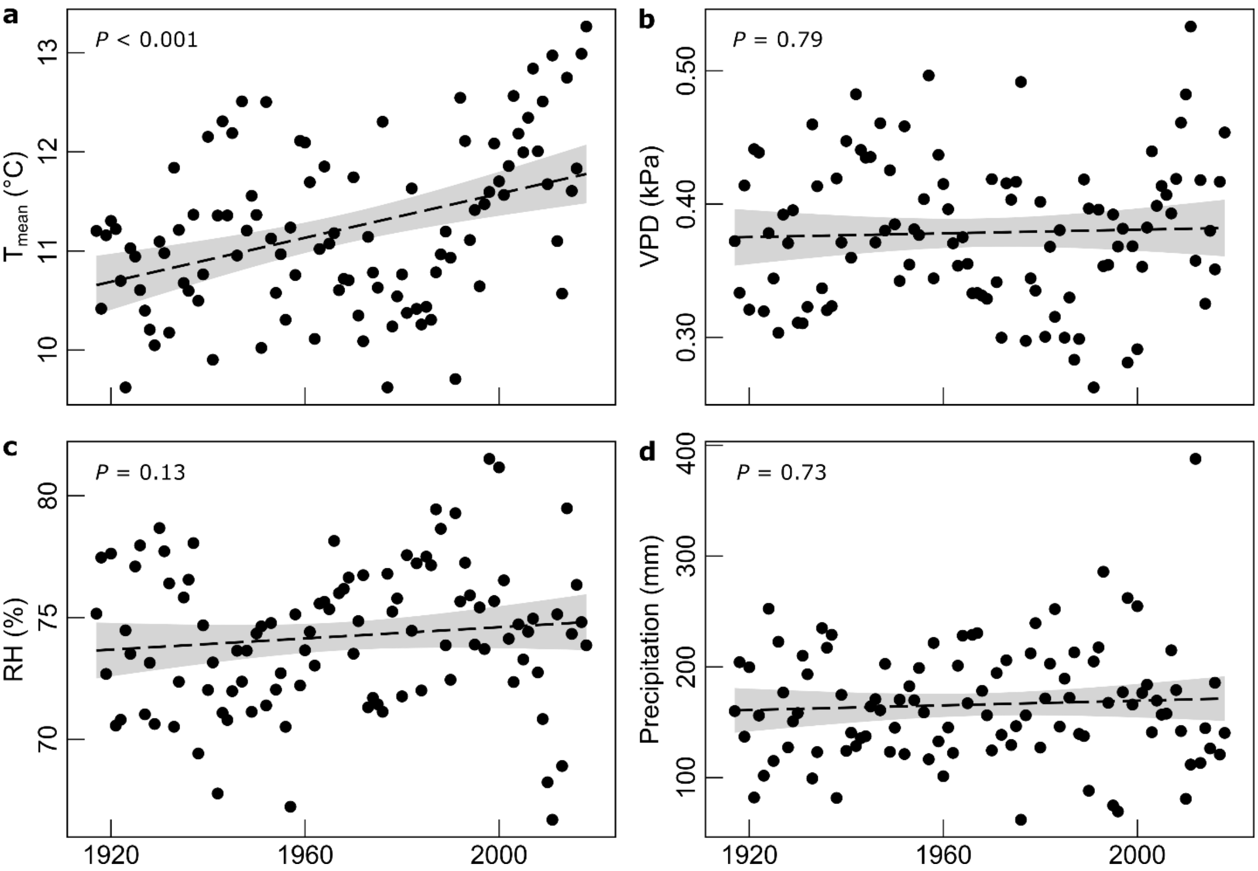

Figure S1. Spring (April-June) meteorological data during the last 100 years. Mean air temperature (**a**), vapour pressure deficit, VPD (**b**), relative humidity of air, RH (**c**) and cumulative precipitation (**d**). The continuous lines and the shadowed area represent the linear regression ± CI95%. Data points correspond to yearly averages of daily values recorded at the Rothamsted Weather Station between April and June. *P*-values correspond to results of Mann-Kendall trend tests.

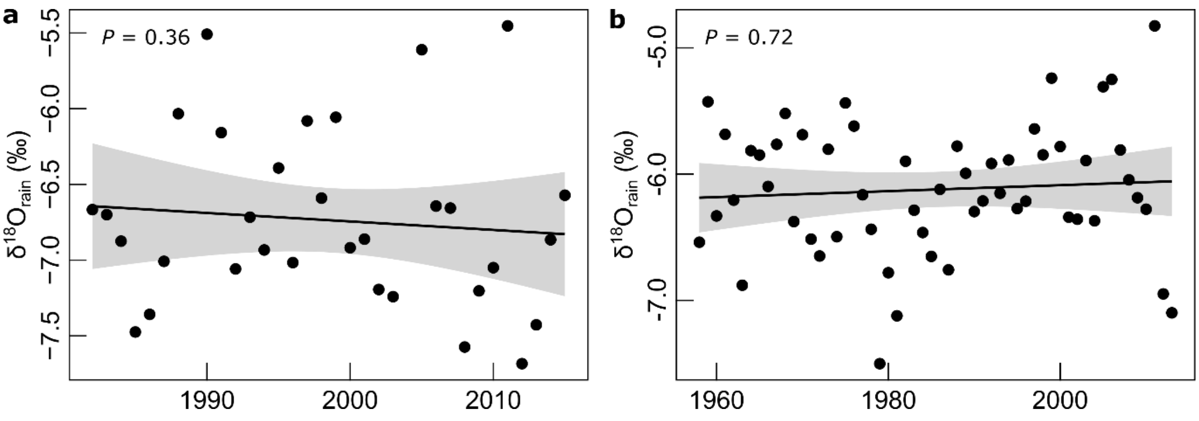

Figure S2. $\delta^{18}O_{rain}$ long-term trends. Long-term trends of $\delta^{18}O_{rain}$ measured at the Wallingford GNIP monitoring station (**a**) or obtained from the outputs of the ECHAM5 model (**b**). Data points are annual averages (Wallingford: 1982-2015; ECHAM5: 1958-2013), calculated from monthly values. The continuous lines and the shadowed areas represent the linear regression ± CI95%. *P*-values correspond to results of Mann-Kendall trend tests.

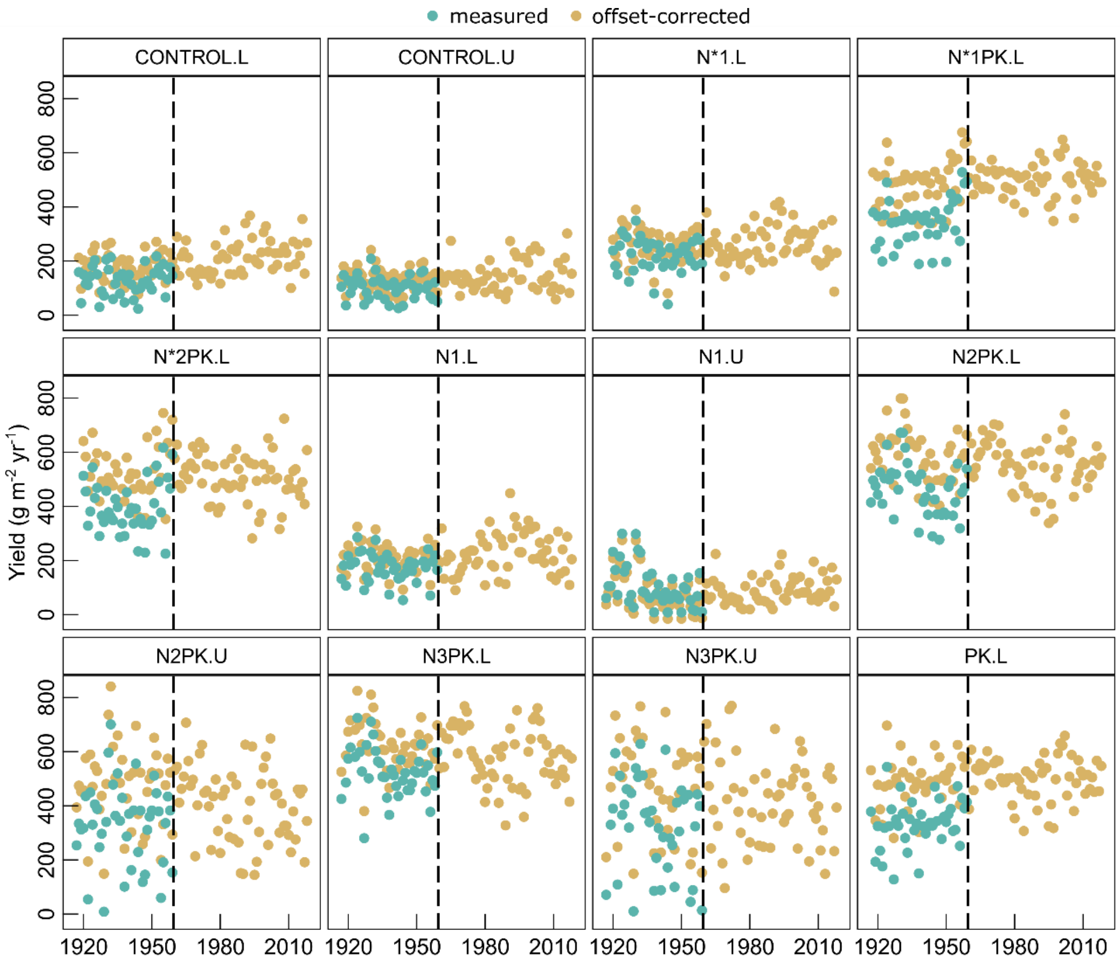

Figure S3. Yields at Park Grass 1917-2018. Total herbage yields (g dry matter m^{-2} yr^{-1}) before (1917-1959) and after (1960-2018). The vertical dashed line indicates the year (1960) when the methodology for yield estimation changed for each individual treatment. Data points before 1960 represent the yields measured with the pre-1960 methodology and the offset-corrected yields.

Table S1. Sample size for all treatments. Number of available yearly samples (*n*) used for the determination of long-term changes in intrinsic water-use efficiency (W_i), yield, $\delta^{18}O_{cellulose}$ and N acquisition. *n* is presented for each treatment, for the ensemble of dicot-rich or grass-rich treatments, and for all treatments, separately for the two analysis periods.

Treatment	Period 1 (1917-1931)				Period 2 (2004-2018)			
	W_i	Yield	$\delta^{18}O_{cellulose}$	N acquisition	W_i	Yield	$\delta^{18}O_{cellulose}$	N acquisition
CONTROL.L	14	14	14	14	15	15	15	15
CONTROL.U	15	15	15	15	12	15	12	13
PK.L	14	14	14	14	13	15	15	15
N*1.L	12	12	11	12	15	15	15	15
N*1PK.L	14	14	13	14	14	15	15	15
N*2.PK.L	12	12	12	12	14	15	14	15
N1.L	12	14	11	13	14	15	15	15
N1.U	15	15	13	15	14	15	15	15
N2PK.L	15	15	13	15	15	15	15	15
N2PK.U	15	15	13	15	15	15	15	15
N3PK.L	13	13	11	13	14	15	15	15
N3PK.U	14	14	12	14	15	15	15	15
Dicot-rich	81	83	78	82	83	90	87	88
Grass-rich	84	84	74	84	87	90	89	90
All treatments	165	167	152	166	170	180	176	178

Table S2. List of publications used for estimation of the average sensitivity of stomatal conductance (g_s) to $\delta^{18}O_{cellulose}$

Reference	Plant material	Experiment type	g_s sensitivity to $\delta^{18}O_{cellulose}$ or $\Delta^{18}O_{cellulose}$ (mol m^{-2} s^{-1} ‰$^{-1}$)	g_s range (mol m^{-2} s^{-1})	Factors for g_s variation
Barbour *et al*., 2000, *Australian Journal of Plant Physiology*	8 cultivars of bread wheat *Triticum aestivum* L.	field	-0.18*	0.47 - 0.63	genetic diversity (cultivars)
Grams *et al*., 2007, *Plant Cell & Environment*	*Fagus sylvatica* L. seedlings<br>*Picea abies* L. seedlings	controlled (phytotrons)	-0.05*<br>-0.04*	0.04 – 0.12<br>0.09 – 0.13	CO_2, O_3, plant-plant competition
Sullivan and Welker 2007, *Oecologia*	*Salix arctica*	field	-0.09†	0.24 – 0.42	Soil temperature<br>Soil water content
Moreno-Gutiérrez *et al*., 2007, *New Phytologist*	10 coexisting species	field	-0.01†	0.07 – 0.18	species
Hirl *et al*., 2021, *New Phytologist*	Temperate grassland ecosystem	field	-0.07†	0.18 – 0.43	seasonal and interannual variation of site conditions
Baca Cabrera *et al*., unpublished,	*Lolium perenne* L.	controlled (phytotrons)	-0.06*	0.06 - 0.18	CO_2

***** g_s sensitivity based on $\delta^{18}O_{cellulose}$ measurements
† g_s sensitivity based on $\Delta^{18}O_{cellulose}$ measurements

Table S3. Pre-1960 yield offset-correction. Yield offset-corrections due to a change of yield determination methodology after 1959 were made for all yield estimates pre-1960 for each individual treatment. The offset-correction was calculated based on the linear regression between c_a and yield in periods pre and post 1960.

Treatment	Average measured yield 1917-1959 (g m^{-2} yr^{-1})	Offset-correction (g m^{-2} yr^{-1})
CONTROL.L	128	+53
CONTROL.U	102	+32
N*1.L	222	+40
N*1PK.L	344	+147
N*2PK.L	399	+128
N1.L	173	+39
N1.U	104	-23
N2PK.L	461	+126
N2PK.U	332	+140
N3PK.L	524	+100
N3PK.U	327	+139
PK.L	336	+153
